# Phylogenetic position of an uncharacterized Brazilian strain of bovine papillomavirus in the genus *Xipapillomavirus* based on sequencing of the L1 open reading frame

**DOI:** 10.1590/S1415-47572010005000091

**Published:** 2010-12-01

**Authors:** Michele Lunardi, Marlise P. Claus, Amauri A. Alfieri, Maria Helena P. Fungaro, Alice F. Alfieri

**Affiliations:** 1 Laboratório de Virologia Animal, Departamento de Medicina Veterinária Preventiva, Universidade Estadual de Londrina, Londrina, PRBrazil; 2 Laboratório de Genética Molecular, Departamento de Biologia Geral, Universidade Estadual de Londrina, Londrina, PRBrazil

**Keywords:** BPV, cattle, cutaneous papillomatosis, putative new BPV type, L1 gene

## Abstract

The use of PCR assays with degenerate primers has suggested the existence of numerous as yet uncharacterized bovine papillomaviruses (BPV). Despite the endemic nature of BPV infections, the identification of BPV types in Brazilian cattle is still only sporadic. However, in a recent analysis of a partial segment of the L1 gene, we observed notable diversity among the BPV types detected. The aim of this study was to determine the phylogenetic position of the previously identified wild strain BPV/BR-UEL2 detected in the state of Paraná in Brazil. Since previous analysis of the partial L1 sequence had shown that this strain was most closely related to BPV type 4, genus-specific primers were designed. Phylogenetic analysis using complete L1 ORF sequences revealed that BPV/BR-UEL2 was related to BPV types classified in the genus *Xipapillomavirus* and shared the highest L1 nucleotide sequence similarity with BPV type 4 (78%). This finding suggests that BPV/BR-UEL2 should be classified as a potential new type of BPV in the genus *Xipapillomavirus*.

Papillomaviruses (PVs) are a highly diverse group of circular double-stranded DNA viruses that can induce epithelial proliferation in a wide range of vertebrate species. In cattle, the bovine papillomavirus (BPV) has been implicated as the casual agent of cutaneous papillomatosis and cancer of the urinary bladder and upper gastrointestinal tract (Campo, 2002).

Recently, based on a comparison of the entire L1 nucleotide sequence of almost all known PVs, the family *Papillomaviridae* was found to consist of 18 genera (*Alphapapillomavirus* to *Sigmapapillomavirus*), each containing a number of species. The different genera show less than 60% identity in the L1 nucleotide sequence, whereas species within a genus share 60%-70% identity. In addition, traditional types within a species show 71%-89% identity in the same gene (De Villiers *et al.*, 2004).

While more than 100 human papillomavirus (HPV) types have been identified, only six BPV types had been described in cattle before 2007. These BPVs were classified in the genera *Deltapapillomavirus* (BPV-1 and -2), *Xipapillomavirus* (BPV-3, -4, and -6) and *Epsilonpapillomavirus* (BPV-5) (De Villiers *et al.*, 2004). In addition, the most recently characterized BPV types were placed in *Epsilonpapillomavirus* (BPV-8) and *Xipapillomavirus* (BPV-9 and -10), with the exception of BPV-7, which belongs to an as yet undesignated PV genus (Ogawa *et al.*, 2007; Tomita *et al.*, 2007; Hatama *et al.*, 2008).

As observed for HPV, PCR assays using degenerate primers that amplify partial fragments of the L1 gene, followed by sequencing, have demonstrated the presence of numerous BPV types in cattle herds from diverse geographical regions. Using the primers FAP59/FAP64 and MY09/MY11, 12 putative new BPV types were detected in teat skin warts and healthy teat skin of cattle from Japan and Sweden (Forslund *et al.*, 1999; Antonsson and Hansson, 2002; Ogawa *et al.*, 2004).

A recent investigation using the strategy above revealed notable diversity among BPV types detected in papillomas of four cattle herds from the state of Paraná in southern Brazil. The study also identified four putative new BPV types designated as BPV/BR-UEL2 to BPV/BR-UEL5 (GenBank accession numbers EU293538 to EU293541, respectively) (Claus *et al.*, 2008). The aim of the current study was to determine the phylogenetic position of BPV/BR-UEL2 in relation to other BPVs.

BPV/BR-UEL2 DNA was isolated from a rice-grain-sized papilloma located in the axillary region of a cow from a dairy cattle herd in Paraná (Claus *et al.*, 2008). The papilloma was homogenized in phosphate-buffered saline (PBS, pH 7.2) and the homogenate (10% w/v) then centrifuged (1500 x *g*, 15 min, 4 °C). An aliquot (250 μL) of the supernatant was treated with lysis buffer [10 mM Tris, 1 mM EDTA, 0.5% Nonidet P40, 1% SDS and 0.2 mg/mL proteinase K (Invitrogen, Life Technologies, USA)], mixed and incubated at 56 °C for 30 min.

DNA was extracted using a combination of the phenol/chloroform/isoamyl alcohol and silica/guanidine isothiocyanate methods (Alfieri *et al.*, 2006). The DNA was eluted in 50 μL of ultrapure (MilliQ^®^) sterile water and stored at -20 °C until used. An aliquot of ultrapure sterile water was included as a negative control in the DNA extraction procedure.

Since FAP sequence analysis of the wild type BPV/BR-UEL2 (GenBank accession number EU293538) has shown that this isolate is most closely (77%) related to BPV type 4, sequences from the L2, L1 and LCR regions of *Xipapillomavirus* representatives (BPV-3, -4 and -6) were aligned and used to design degenerate primers to obtain the entire L1 nucleotide sequence.

Combinations of the two primer sets (L2Bf/L1Br and L1Bf/LCRBr) and the previously described FAP primer pair were tested (see Table 1 for primer features) (Forslund *et al.*, 1999). In addition, the same primer sets were also tested on DNA samples known to harbor BPV type 6, a *Xipapillomavirus* representative. Sequence alignment and primer design were done using the CLUSTAL W Multiple Alignment program and Gene Runner v 3.05 (Hastings Software Inc., Hastings, NY), respectively (Thompson *et al.*, 1994).

The PCR reactions contained 2.5 μL of extracted DNA, 20 pmol of each primer, 200 μM of each dNTP, 2.5 U of Platinum *Taq* DNA polymerase (Invitrogen), 1 x PCR buffer (20 mM Tris-HCl, pH 8.4 and 50 mM KCl), 1.5 mM MgCl_2_ and ultrapure sterile water in a final volume of 25 μL. The reactions were run in a PTC-200 thermocycler (MJ Research Co., USA) using the following times and temperatures: 10 min at 94 °C followed by 40 cycles of 1 min at 94 °C, 1 min at an optimum temperature for primer annealing, 1 min at 72 °C, and a final extension of 10 min at 72 °C. The annealing temperatures for primer pairs L2Bf/FAP64, L2Bf/L1Br and L1Bf/LCRBr were 50 °C, 54 °C and 57 °C, respectively. The amplified products were analyzed by electrophoresis in 1.5% agarose ethidium bromide stained gels in TBE buffer, pH 8.4 and examined under UV light.

Initially, all PCR products were purified using a PureLink quick gel extraction kit (Invitrogen) and then cloned using TOPO TA cloning kit for sequencing (Invitrogen), according to the manufacturer's instructions. The inserts from two clones selected for each PCR amplicon were then sequenced using DYEnamic ET dye terminator cycle sequencing kit (GE Healthcare, Little Chalfont, UK) with M13 forward and reverse primers in a MegaBACE 1000/Automated 96 Capillary DNA sequencer (GE Healthcare), according to the manufacturer's instructions. The sequences obtained were examined with the PHRED application for quality analysis of chromatogram readings. The sequences were accepted if base quality was ≥ 20. The consensus sequence was determined using CAP3 software and the sequence identity was verified against all sequences deposited in GenBank using the BLAST application. The L1 ORF of Brazilian wild type BPV was predicted based on analysis with the ORF Finder tool. The alignment and degree of similarity among sequences at the nucleotide and amino acid levels were determined using BIOEDIT v 5.0.9 software (Hall, 1999). The phylogenetic tree was constructed using MEGA v. 3.1 software (Kumar *et al.*, 2004) and the neighbor-joining method with the Kimura two-parameter distance estimate (Kimura, 1980). Bootstrap support values were determined for 1000 replications.

The first L1 segment of the Brazilian wild strain BPV was obtained using a semi-nested PCR assay (SN-PCR) with the primer pair L2Bf/FAP64 in the first round and the primer pair L2Bf/L1Br in the second round, which yielded an amplicon of 435 bp. Use of the FAP59/FAP64 (475 bp) and L1Bf/LCRBr (1128 bp) primer sets allowed amplification of the remaining portions of the same gene (Figure 1).

A consensus sequence of 1804 nt (GenBank accession number GQ471901) spanning nt 5385 to nt 7184 of BPV-4 was obtained with the L2Bf/L1Br, FAP59/FAP64 and L1Bf/LCRBr overlapping amplicons of the BPV/BR-UEL2 isolate. Phylogenetic analysis using complete L1 ORF sequences revealed that the BPV/BR-UEL2 isolate was related to BPV types in the genus *Xipapillomavirus* (Figure 2). In addition, pairwise DNA sequence alignments showed that this isolate shared the highest L1 nucleotide sequence similarity with BPV type 4 (78%), which again suggested its classification in the genus *Xipapillomavirus*; at the amino acid level, the identity with BPV-4 was 83.6%. Finally, DNA samples known to contain BPV-6 were amplified and the three overlapping amplicons (L2Bf/FAP64, FAP59/FAP64 and L1Bf/LCRBr) were obtained in all of the samples tested.

The L1 nucleotide sequence of our isolate showed 71%-78% identity with *Xipapillomavirus* representatives whereas with representatives of other genera the similarity was 55.6-55.9%. A phylogenetic analysis based on amino acid sequence alignments yielded a tree with a topology very similar to that based on nucleic acid sequence alignments (data not shown).

Compared with other BPV genera, *Xipapillomavirus* contains a great diversity of BPV types, as shown by studies in which several putative new BPV types have been identified, *e.g.*, BAA-1, BAPV-3, -8, -9 and -10 and BPV/BR-UEL3 (Antonsson and Hansson, 2002; Ogawa *et al.*, 2004; Claus *et al.*, 2008).

BPV type 4 has traditionally been associated with cattle alimentary tract tumors (Campo *et al.*, 1994; Borzacchiello *et al.*, 2003). However, our Brazilian BPV isolate was detected in a skin wart located in the axillary region of a dairy cow and was thus associated with cutaneous papillomatosis.

Despite the similarity with BPV-4, the L1 ORF-encoded protein of this Brazilian isolate consisted of 532 amino acids, similar to the L1 protein of BPV-9, a newly described *Xipapillomavirus* type, which contains 531 amino acids. In contrast, the BPV-4 L1 ORF codes for a 506 amino acid protein. ORF analysis of the consensus sequence revealed an overlap between L2 and L1, also seen in BPV-9 and -10, which differed from that observed in BPV-4.

In contrast to our putative novel BPV type, the upstream fragment of the L1 gene of BPV-6 could be obtained by PCR using the L2Bf/FAP64 primer set whereas the subsequent L1 segments were obtained using the same primer pairs as those used for the BPV/BR-UEL2 isolate (data not shown). The specificity of the amplified products was confirmed for one of these samples by analyzing the resulting consensus sequence.

The strategy developed in this study involved the use of three pairs of degenerate primers, two of them specifically designed to amplify the L1 gene of certain *Xipapillomavirus* representatives. This approach was very useful since it allowed us to easily and unambiguously obtain the entire L1 gene sequence and determine the correct phylogenetic position of an uncharacterized BPV type.

Despite the relatively common occurrence of BPV infections in Brazil, the identification of BPV types in cattle herds is still sporadic. In addition, the few studies done have usually involved the use of type-specific primers and have determined the presence of BPV types 1 and 2 (Dos Santos *et al.*, 1998; De Freitas *et al.*, 2003; Wosiacki *et al.*, 2005, 2006). Recently, use of the FAP59/FAP64 primer pair allowed the identification of previously described BPV types (BPV-1, -2, -6 and -8) and four putative new BPV types in skin warts of cattle from Paraná (Claus *et al.*, 2007, 2008, 2009a,b,c).

**Figure 1:**
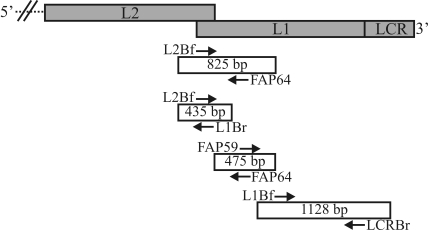
Schematic diagram showing the relative positions of overlapping PCR fragments in the L2, L1, and LCR regions of the BPV/BR-UEL2 isolate. The length of each amplicon is indicated in the white box while the primer sets are shown as arrows.

**Figure 2:**
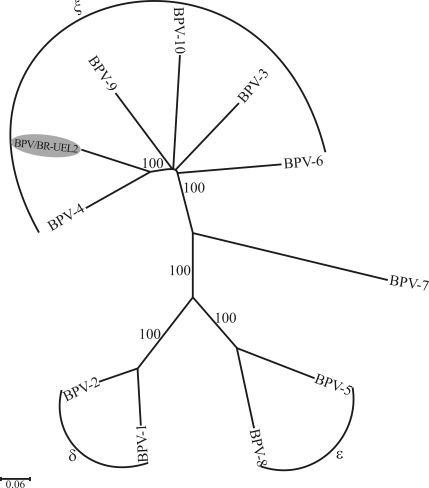
Neighbor-joining phylogenetic tree of L1 ORFs (nt) of BPVs, including that of the BPV/BR-UEL2 isolate (indicated by shading). The tree is divided into the previously determined genera *Deltapapillomavirus* (BPV-1 and -2), *Epsilonpapillomavirus* (BPV-5 and -8), *Xipapillomavirus* (BPV-3, -4, -6, -9 and -10) and an undesignated PV genus (BPV-7). The numbers at the internal nodes represent the bootstrap support values determined for 1000 replications.

In Brazil, cattle diseases caused by BPV, such as chronic enzootic hematuria, cancer of the upper gastrointestinal tract and teat papillomatosis, have prevented the economic exploitation of livestock in specific geographic areas. However, few studies have sought to characterize these pathogens and those aiming at developing means of controlling and treating these diseases have been neglected in Brazil. The importance of the molecular characterization of wild type BPVs isolated from Brazilian cattle rests with the fact that, as in humans, the identification of viral types associated with particular clinical manifestations and the definition of the most prevalent types could spur the development of prophylactic and therapeutic vaccines as means of controlling diseases in the field.

## Figures and Tables

**Table 1:** Sequences and features of primers used in the polymerase chain reactions.

Primer	Genomic region targeted	Polarity	Sequence^1^	Nucleotide positions^2^	Degree of degeneracy
L2Bf	L2	+	5'GTIAARYTITTYATHAAYGAYGC3'	5385-5407	96
FAP59^3^	L1	+	5'TAACWGTIGGICAYCCWTATT3'	5729-5749	8
L1Br	L1	-	5'AASACTCTGAATTGACTGCC3'	5794-5813	2
L1Bf	L1	+	5'GRGAGCAYTGGGAYAAAG3'	6089-6106	8
FAP64^3^	L1	-	5'CCWATATCWVHCATITCICCATC3'	6175-6197	36
LCRBr	LCR	-	5'CWRCATTTTATTKSSAASATTC3'	7181-7202	64

^1^Degenerate nucleotides: I = inosine; R = G, A; Y = T, C; H = A, T, C; W = A, T; S = G, C; V = G, A, C; K = G, T. ^2^Relative position in the BPV-4 genome. ^3^Forslund *et al.* (1999).
